# BUILDING BRIDGES: AN AGE-FRIENDLY CONFERENCE FOR THE GEROPSYCHOLOGY WORKFORCE

**DOI:** 10.1093/geroni/igac059.298

**Published:** 2022-12-20

**Authors:** Brian Carpenter, Jennifer Moye, Joseph Dzierzewski

## Abstract

As in other disciplines, there is a high demand for psychologists who have specialized training in aging, but the demand far outstrips the supply, with only 1% of clinical/counseling psychologists identifying aging as their area of focus. The field of geropsychology has held a series of training conferences, the last in 2006, to define a training model and aging-related competencies. Fifteen years later, the field gathered again for a conference focusing specifically on the pipeline, with the goals of 1) understanding the recent shortage of applicants for positions in academic settings, 2) addressing underrepresentation of individuals from diverse racial and ethnic backgrounds across clinical and academic geropsychology, and 3) implementing concrete solutions. In this symposium we describe our two-day, four-hour virtual national conference held in 2021, attended by more than 150 psychologists nationwide, including the structure and outcomes of the conference and the progress of several ongoing working groups. The first paper summarizes quantitative and qualitative findings of a pre-conference survey on perceptions of the geropsychology workforce. The second paper describes a career pathways webinar aimed at graduate students, interns, and fellows to attract students to diverse careers in aging. The third paper describes discussions about the impact of the pandemic on geropsychology training. The fourth paper presents a survey and efforts of a working group focused on post-licensure training. This presentation offers a possible model for others considering ways to galvanize interest and training in aging.

